# Macrothrombocytopenia of Takenouchi-Kosaki syndrome is ameliorated by CDC42 specific- and lipidation inhibitors in MEG-01 cells

**DOI:** 10.1038/s41598-021-97478-y

**Published:** 2021-09-09

**Authors:** Etsuko Daimon, Yukinao Shibukawa, Suganya Thanasegaran, Natsuko Yamazaki, Nobuhiko Okamoto

**Affiliations:** 1 Department of Molecular Medicine, Research Institute, Osaka Women’s and Children’s Hospital, 840 Murodo-cho, Izumi, Osaka 594-1101 Japan; 2 Department of Medical Genetics, Research Institute, Osaka Women’s and Children’s Hospital, 840 Murodo-cho, Izumi, Osaka 594-1101 Japan

**Keywords:** Diseases, Molecular medicine

## Abstract

Macrothrombocytopenia is a common pathology of missense mutations in genes regulating actin dynamics. Takenouchi-Kosaki syndrome (TKS) harboring the c.191A > G, Tyr64Cys (Y64C) variant in Cdc42 exhibits a variety of clinical manifestations, including immunological and hematological anomalies. In the present study, we investigated the functional abnormalities of the Y64C mutant in HEK293 cells and elucidated the mechanism of macrothrombocytopenia, one of the symptoms of TKS patients, by monitoring the production of platelet-like particles (PLP) using MEG-01 cells. We found that the Y64C mutant was concentrated at the membrane compartment due to impaired binding to Rho-GDI and more active than the wild-type. The Y64C mutant also had lower association with its effectors Pak1/2 and N-WASP. Y64C mutant-expressing MEG-01 cells demonstrated short cytoplasmic protrusions with aberrant F-actin and microtubules, and reduced PLP production. This suggested that the Y64C mutant facilitates its activity and membrane localization, resulting in impaired F-actin dynamics for proplatelet extension, which is necessary for platelet production. Furthermore, such dysfunction was ameliorated by either suppression of Cdc42 activity or prenylation using chemical inhibitors. Our study may lead to pharmacological treatments for TKS patients.

## Introduction

Cdc42 belongs to the Ras superfamily of small GTPase proteins and is implicated in a variety of biological activities to regulate proteins interacting with the actin cytoskeleton such as p21-activated kinase (PAK) and Wiskott-Aldrich syndrome protein (WASP)^1–3^. Cdc42 cycles between a GTP- and a GDP-bound state and signals its effectors when in the active GTP-bound state. The state of activity is controlled by three different classes of regulators: guanine nucleotide exchange factors (GEF), GTPase-activating proteins (GAP) and guanine nucleotide dissociation inhibitors (GDI)^4^. Recruitment to a specific membrane is necessary for its activation and requires the modification of the C-terminal end with a geranylgeranyl anchor^5^. It was recently reported that missense variants in CDC42 underlie a heterogeneous group of phenotypes characterized by growth retardation, facial dysmorphism, and neurodevelopmental, immunological and hematological anomalies that resemble Noonan syndrome^6^.

Macrothrombocytopenia is associated with pathogenic variants in genes that regulate actin dynamics, such as Aip1/Wdr1, which are partners of cofilin^7^, ACTN1 and FLNA (encode actin-crosslinking protein, actinin-1 and Filamin A, respectively)^8,9^ and DIAPH1 (encode actin nucleation and polymerization)^10^. Although there are many previous studies, no therapeutic strategy for macrothrombocytopenia has been developed. Recently, we and others identified a germline heterozygous variant in CDC42 implicated in human disorder^6,11–13^. Takenouchi-Kosaki syndrome (TKS) harboring the c.191A > G, Tyr64Cys variant (Y64C) causes intellectual and growth delay, dysmorphism, lymphedema in the lower extremities, camptodactyly and macrothrombocytopenia^6,11,12,14,15^. When the patients are critically ill, hemorrhagic diathesis is life-threatening. The expression of Y64C in neurons impairs their morphology and migration both in vitro and in vivo in a mouse model^16^. These defects are likely responsible for the pathology of psychomotor delay in TKS patients. However, the molecular mechanisms underlying the clinical features remain to be clarified.

Platelets are produced from megakaryocytes (MK) that undergo complex maturation processes, including DNA replication without cell division, and extension of cytoplasmic protrusions termed proplatelet formation^17–19^. Mice lacking Cdc42 specifically in MK exhibit macrothrombocytopenia and impaired platelet function^20^. PAK plays an essential role in demarcation membrane system formation and affects the subsequent process of proplatelet differentiation^21^. MK polarization and transendothelial thrombopoiesis are controlled by GP1b one of the downstream effectors of Cdc42^22^. Palazzo et al. reported that Cdc42 regulates proplatelet formation through N-WASP^23^. These studies suggested the importance of Cdc42 in MK maturation and thrombopoiesis. In the present study, we analyzed the function of Y64C using the HEK293 cell line and evaluated its effects on thrombopoiesis using the MEG-01 cell line, which produces platelet-like particles (PLP) in vitro^24^. Y64C facilitated membrane localization through the dysfunction of Rho-GDI association. Y64C also suppressed both the extension of cytoplasmic protrusions and PLP production, although it promoted polyploidy. Furthermore, the dysfunction in proplatelet formation processes, including cytoplasmic extension and PLP production, was ameliorated by either suppression of Cdc42 activity or cellular localization using chemical inhibitors, which eventually led to the improvement of PLP production by Y64C to the same extent as wild-type Cdc42 (WT).

## Materials and methods

### Cell culture, Pasmid and lentivirus vector construct

HEK293 cells were provided by ATCC (Baltimore, MD) and maintained in DMEM supplemented with 10% FCS. The MEG-01 cell line was provided by the RIKEN BRC (Ibaragi, Japan) through the National Bio-Resource Project of MEXT, Japan. Cells were continuously cultivated in RPMI 1640 medium (FUJIFILM Wako Pure Chemical, Osaka, Japan) supplemented with 10% FCS (Sigma-Aldrich, St. Louis, Missouri, MO), 50 IU/ml of penicillin and 50 μg/ml of streptomycin at 37 °C in a humidified atmosphere of 5% CO_2_. Human cdc42 cDNA was amplified from the random-primed in-house cDNA library of HEK293 cells and inserted into the XhoI/BamHI site of pENTR/flag to generate N-terminal Flag-tagged cdc42, or a XhoI/BamHI site of EYFP-C1 (Clontech, Mountain View, CA) to generate EYFP-cdc42. Site-directed mutagenesis was performed using a KOD-Plus Mutagenesis kit (TOYOBO, Osaka, Japan) according to the manufacturer’s protocol. Recombinant lentiviruses provided by RIKEN BRC were produced by transient transfection according to reported protocols^25^. Briefly, sub-confluent 293T cells were co-transfected with the self-inactivating vector pCAG-HIVgp and pCMV-VSV-G-RSV-Rev by calcium phosphate precipitation. The medium was changed 18 h later and the recombinant lentiviral particles were harvested after an additional 48-h incubation.

### Co-immunoprecipitation (IP) assay, in gel digestion and peptide mass fingerprint

Cells were lysed in co-IP buffer (0.1% NP-40, 0.3% Triton X-100, 150 mM NaCl, 5 mM EDTA, 1 mM aprotinin, 1 mM PMSF and 20 mM Tris–HCl, pH 7.2) and then centrifuged at 15,000 × *g* for 15 min. Supernatants were collected and protein concentrations were measured using the Bradford method. Equal amounts of proteins were incubated with anti-Flag M2–agarose beads (Sigma-Aldrich) at 4 °C for 90 min. Immunoprecipitants were washed with co-IP buffer 3 times and boiled in SDS sample buffer for 5 min. Supernatants were loaded on a 10% SDS-PAGE gel and developed by silver-staining. Regarding in gel digestion and peptide mass fingerprinting, gel pieces containing the band showing differential expression were cut and washed in 300 μl of CH_3_CN for 30 min. After drying, they were rehydrated in 100 μl of reduction buffer (10 mM DTT and 100 mM NH_4_HCO_3_) and left standing at 56ºC for 1 h. The gel pieces were incubated in 100 μl of 100 mM NH_4_HCO_3_ containing 50 mM iodoacetamide at room temperature for 45 min. The gel pieces were washed and dehydrated inCH_3_CN twice, and the dried gel pieces were rehydrated on ice in 50 mM Tris–HCl pH 8.0 containing lysylendopeptidase (WAKO, Osaka, Japan) and modified trypsin (Promega, Madison, WI) for 45 min. The digested peptides collected from the supernatant were evaporated to dryness in a vacuum centrifuge. Resulting peptides were absorbed onto Zip Tip C18 (Merck Millipore, Burlington, MA) and eluted with 50% CH_3_CN and 0.1% trifluoroacetic acid (TFA). Equal amounts of the desalting peptides solution and a sample matrix (saturated α-cyano-4-hydroxycinnamic acid dissolved in 50% CH_3_CN and 0.1% TFA) for matrix-assisted laser desorption/ionization (MALDI) were mixed on the sample target for mass spectrometry (MS). Peptide mass fingerprinting was carried out using Mascot search for the peptide masses obtained by a Voyager DE-Pro time-of-flight mass spectrometer (Applied Biosystems, Foster city, CA).

### Western blotting

Cells were cultured in serum-free medium overnight and stimulated with medium containing 10 ng/ml of EGF or 50 ng/ml of PMA for 2 min and co-immunoprecipitation assay were performed. Supernatants were loaded on a 10% SDS-PAGE gel and then transferred to PVDF membranes. The membrane was incubated with primary and secondary antibodies for 1 h each, and detection was performed using an ECL kit (GE Healthcare) according to the manufacturer’s instructions.

### GTPase activity assay

Cells were cultured in serum-free medium overnight and stimulated with medium containing 10 ng/ml of EGF for 2 min. Cells were lysed and the particulate fraction was removed by centrifugation. Active Cdc42 was pulled-down using the Rac1/Cdc42 activation assay kit (Merck Millipore) according to the manufacturer’s instructions. The amount of the GTP-bound form of Cdc42 was detected by Western blotting using anti-Cdc42 or anti-GFP antibodies.

### Cell fractionation

Cells were lysed in hypotonic buffer (10 mM KCl, 1.5 mM MgCl_2_ 0.1 mM EDTA, 1 mM aprotinin, 1 mM PMSF and 10 mM Hepes, pH 7.9) and homogenized. Cell homogenates were centrifuged at 2,000x*g* for 5 min. The supernatants were transferred to a new tube and additionally centrifuged at 55,000 × *g* at 4 °C for 30 min to separate the cytosolic and microsomal membrane fractions. The pellets containing the microsomal membrane fraction were lysed in RIPA buffer and centrifuged at 15,000 rpm at 4 °C for 10 min and the soluble microsomal membrane fractions were collected.

### Megakaryocytic differentiation

MEG-01 cells (3 × 10^5^ cells/ml) were seeded in 6-well plates and incubated with medium containing 2 mM valproic acid (VPA, FUJIFILM Wako Pure Chemical) for up to 12 days with medium containing 2 mM VPA added on the 7th day. Cdc42 inhibitors ML141 (Merck Millipore), R-ketorolac (Sigma-Aldrich) and geranylgeranyl transferase inhibitor GGTI-298 (CAYMAN CHEMICAL, Ann Arbor, MI) were added once every 3–4 days. After differentiation, attached cells were harvested by scraping and combined with floating cells. Collected cells were spin down at 70xg for 10 min and the supernatants were further centrifuged at 1,500xg for 10 min to harvest PLP. Differentiated cells or PLP were fixed in 2% paraformaldehyde (PFA)/PBS for 30 min at 4 °C and then washed with PBS. Pellets were suspended in 0.3 ml of PBS containing sodium azide and stored at 4 °C for analysis by flow cytometry.

### Fluorescence-activated cell sorting (FACS) analysis and staining procedure

Fixed cells or PLP were analyzed by flow cytometry using anti-CD61-PE (Invitrogen, Carlsbad, CA) antibodies. The PLP production capacity was defined using flow count beads (Beckman Coulter, Brea, CA) as an internal control. In total, 5 × 10^4^ flow count beads were added to each sample. The number of YFP^+^CD61^+^ events with forward/side scatter (FSC/SSC) properties as human peripheral blood platelets per 2,000 units of flow count were enumerated. The number of PLP per cell was calculated by the following equation: PLP density (event/μl) = the concentration of flow count x number of PLP/number of beads x the volume of flow count/the volume of PLP suspension. Absolute number of PLP per YFP- and CD61-double-positive cells = PLP density x total PLP volume / number of plated YFP-positive cells. PLP size was recorded as the GeoMean of FSC in FACS analysis.

### Immunocytochemistry, filopodia formation, nuclear lobulation, protrusions and line scan analysis

Cells were seeded at 1 × 10^4^ cells on an 8-well slide chamber under differentiation conditions for 5 to 7 days. After fixation with 4% PFA/PBS for 10 min and permeabilization with 0.2% Triton X-100 for 1 min, cells were incubated with monoclonal mouse anti β-tubulin (Sigma) at 4 °C overnight. The structure of intercellular β-tubulin and F-actin was visualized using a Leica TCS SP8 confocal microscope by triple-labeling with Alexa 563-coupled donkey anti-mouse secondary antibody (Thermo Fisher, Waltham, MA), Alexa 633-coupled phalloidin (Thermo Fisher), and DAPI respectively. The number of filopodia per cell and cells with nuclear lobulation were counted in at least 50 cells for each cell type. For nuclear area and protrusion analysis, images were processed using Image Pro plus software (Media Cybernetics, Rockville, MD). Fluorescent line scans of β-tubulin and F-actin were performed using the Leica TCS SP8 software along the vertical line in protrusions for YFP and YFP-WT cells and marginal fibrillary structures for YFP-Y64C and YFP-G12V. Line scans were conducted for at least 20 cells in each cell type.

### Ploidy analysis

Differentiated MEG-01 cells were harvested and suspended in DNA staining solution containing 0.05% saponin, 10 μg/ml of ribonuclease A and 50 g/ml of propidium iodide (PI). After incubation for 60 min at 37 °C, cells were washed twice and resuspended in PBS containing 1 mM EDTA and 1% BSA for use in FACS analysis.

### Statistical analysis

All experiments were repeated at least three times independently. Data are presented as the mean ± SEM. Significant differences between groups were analyzed by the Bonferroni/Dunn’s multiple comparison test using Excel statistics. In the analysis of nuclear lobulation and the roundness index (RI), data are presented as the mean ± SD and were analyzed by the Tukey–Kramer multiple comparison test. Values of *P* < 0.05 were considered to be significant and *P* < 0.01 represents sufficient significance.

## Results

### The Y64C variant localizes to the plasma membrane and possesses higher activity

**Figure 1:**
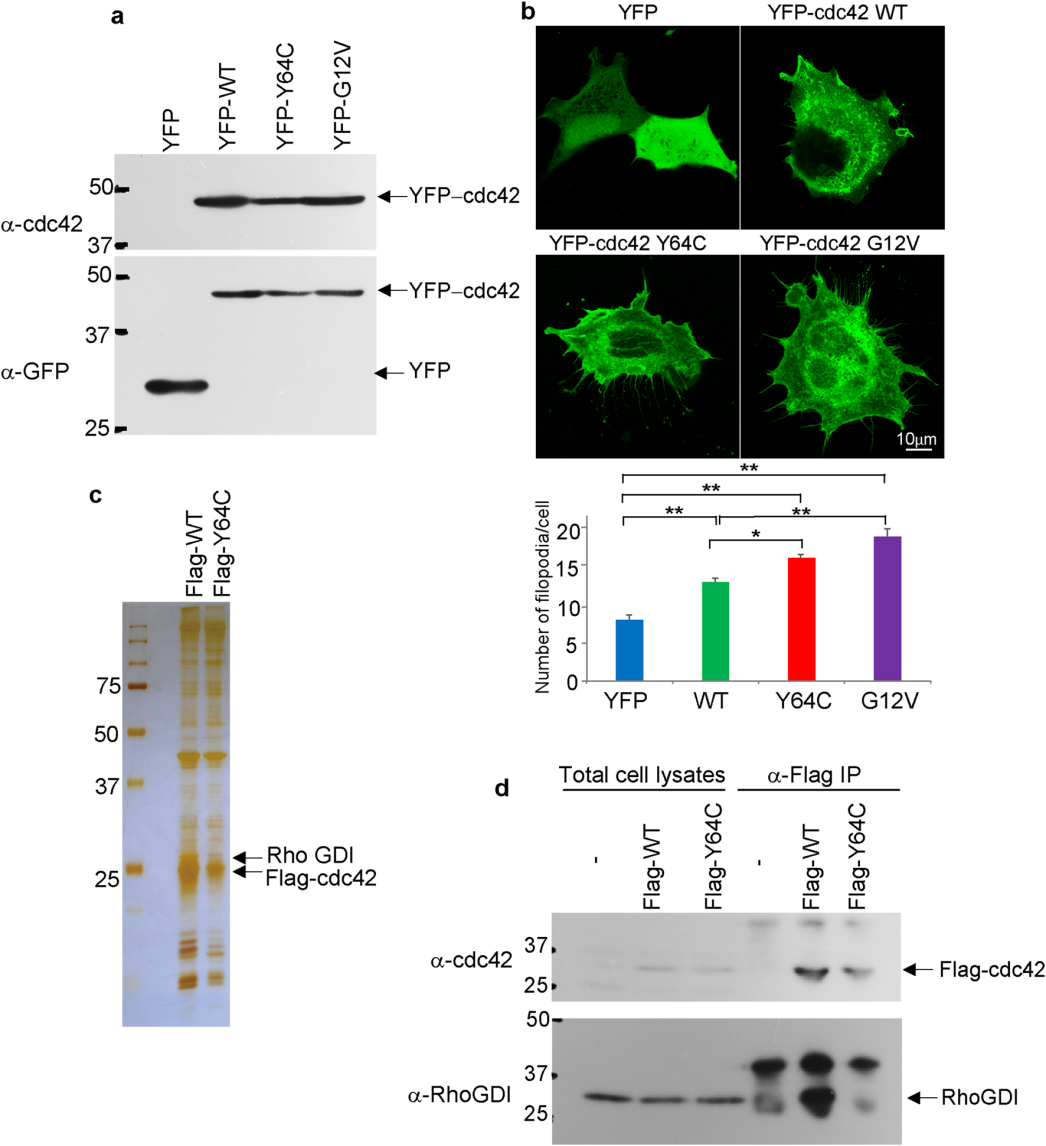
Cdc42-Rho-GDI association was suppressed by Y64C expression. (**a**) Total cell lysates from HEK293 cells transiently expressing EYFP-tagged WT or Cdc42 variants were subjected to SDS-PAGE followed by Western blotting using the indicated antibodies. (**b**) Upper panel; Cellular morphology of EYFP-Cdc42s expressing HEK293 cells. Lower panel; The number of filopodia per cell was counted in at least 50 cells and quantified. Data are the mean ± SEM *P < 0.05, **P < 0.01 (**c**) Flag-tagged WT or Y64C was introduced into HEK293 cells by lentivirus and immunopurified using anti-Flag agarose. The eluates were subjected to SDS-PAGE followed by silver staining. The bands (arrows) were subjected to in-gel digestion followed by peptide mass fingerprinting. (**d**) Co-IP assay was performed using anti-Flag antibody and Cdc42-Rho-GDI association was confirmed by Western blotting using the indicated antibodies. Full length blots and a gel are presented in Supplemental Fig. 1a–c.

First, to investigate whether Y64C affects cellular morphology, YFP-tagged WT, Y64C, or the constitutively active form (CA; G12V) of Cdc42s were introduced into HEK293 cells using lentivirus. An equal amount of YFP-tagged WT or variant Cdc42 was expressed in HEK293 cells (Fig. 1a and Supplemental Fig. 1a for full blots). The formation of filopodia was significantly greater in YFP-tagged Y64C and G12V-overexpressing cells than YFP-control and YFP-tagged WT (Fig. 1b). This suggested that YFP-tagged Y64C mutation affects Cdc42 activity. Next, to identify altered Cdc42-associated molecules between WT and Y64C, Flag-tagged Cdc42-associated molecules were purified from HEK293 cell lysate using anti-Flag antibody. SDS-PAGE followed by silver staining and peptide mass fingerprinting revealed that Y64C did not associate with Rho GDP-dissociation inhibitor (Rho-GDI) (Fig. 1c and Supplemental Fig. 1b for full blots). The co-immunoprecipitation assay confirmed that Y64C did not bind Rho-GDI (Fig. 1d and Supplemental Fig. 1c for full blots).

**Figure 2:**
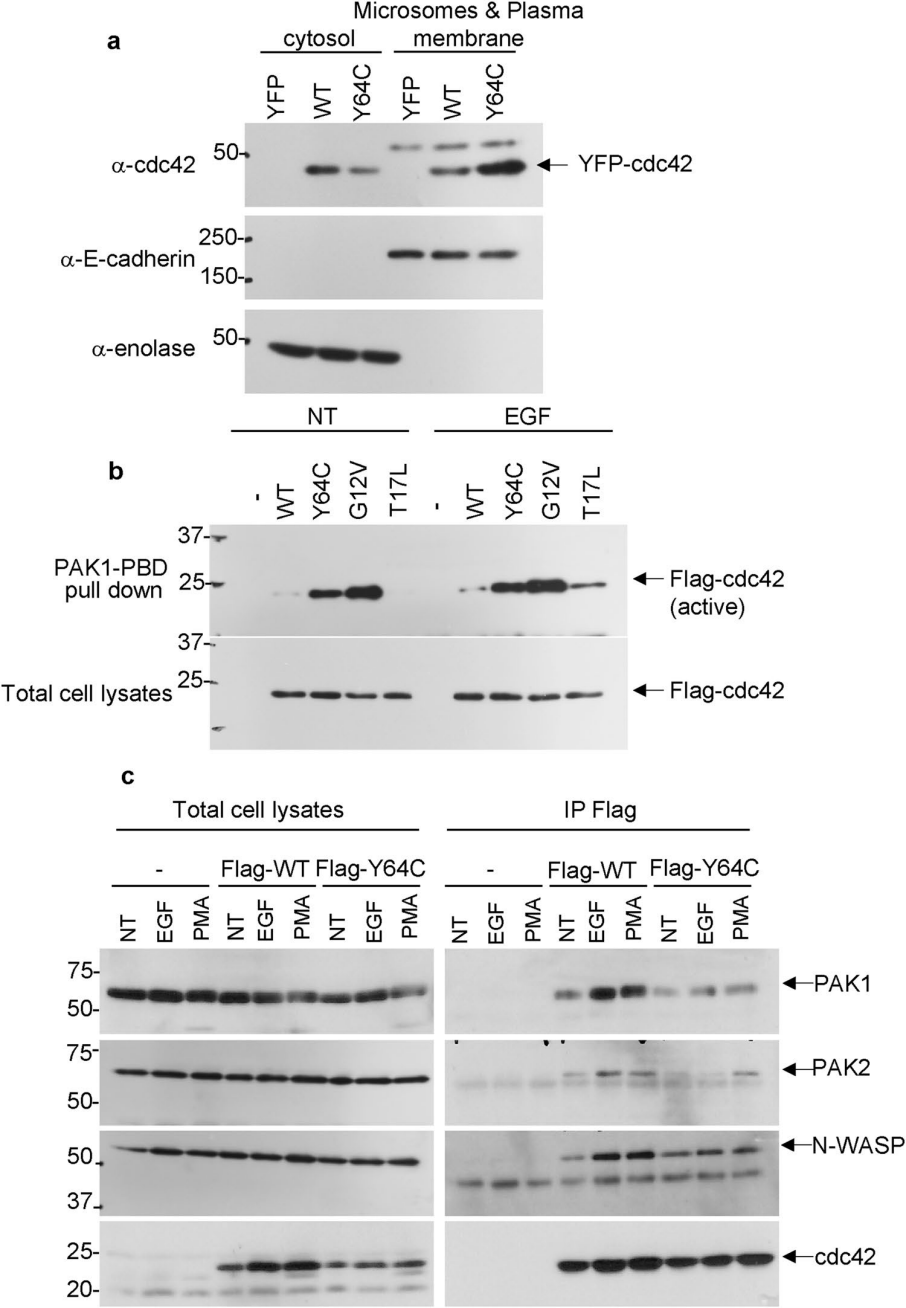
Y64C facilitated both membrane localization and activity, and affected downstream signaling. (**a**) After EYFP-tagged Cdc42 was introduced into HEK293 cells, cytosolic and membrane (microsome and plasma membrane) fractions were separated by ultracentrifugation. Each fraction was subjected to SDS-PAGE followed by Western blotting using the indicated antibodies. (**b**) EYFP-tagged WT or Y64C was introduced into HEK293 cells and equal amounts of proteins were subjected to pull-down assay using PAK-RBD to isolate GTP-bound Cdc42. Cell lysates were reacted with anti-cdc42 antibody. (**c**) Flag-tagged WT or Y64C was recovered using anti-Flag agarose and co-IP assay was performed using the indicated antibodies. Full length blots are presented in Supplemental Fig. 1d–f.

Rho-GDI binds to the switch domain of Cdc42, and inhibits both GDP dissociation and GTP hydrolysis^26^. Inactive Cdc42 is maintains cytosolic localization by associating with Rho-GDI^27^. 64Tyr located in the switch 2 domain is expected to interact with Rho-GDI based on the crystal structure^4^. We hypothesized that Y64C accelerates membrane localization compared with WT. To test this hypothesis, HEK293 cells were fractionated by ultracentrifugation and Western blotting was performed. Fractionation was confirmed by the detection of E-cadherin and enolase expression as markers of the membrane and cytosol, respectively. As shown in Fig. 2a and Supplemental Fig. 1d for full blots, Y64C was enriched in microsomes and plasma membrane. Next, to investigate whether Y64C affects the GTP-bound (active) form of Cdc42, a pull-down assay was performed using beads coupled with PAK-PBD. WT activation was confirmed after EGF exposure; however, Y64C exhibited higher activity in the presence and absence of EGF stimulation, similar to CA (Fig. 2b and Supplemental Fig. 1e for full blots). This suggests that the Y64C mutation causes markedly altered activity, consistent with the previous report that this mutation exhibits robust GAP insensitivity^6,16^.

Several mutations of Cdc42 were found to impact the interaction with effectors in vitro^6^. We next investigated whether Y64C affects downstream signaling. Co-IP assays were performed to assess the interaction between Cdc42s and its effectors, including Pak1/2 and N-WASP. Western blot analysis revealed that the interaction between Pak1/2 and Y64C was weaker than that with WT under basal conditions (Fig. 2c and Supplemental Fig. 1f for full blots). Moreover, Y64C responded to EGF or PMA (Fig. 2c Supplemental Fig. 1f for full blots). In the absence of ligand stimulation, Y64C also bound N-WASP similarly to WT, but its binding was not increased by ligand exposure or Pak1/2 (Fig. 2c). This suggested that Y64C does not respond to stimulation and affects downstream signaling, which controls the cellular processes.

### The Y64C variant accelerates nuclear polyploid formation in MEG-01 cells

**Figure 3:**
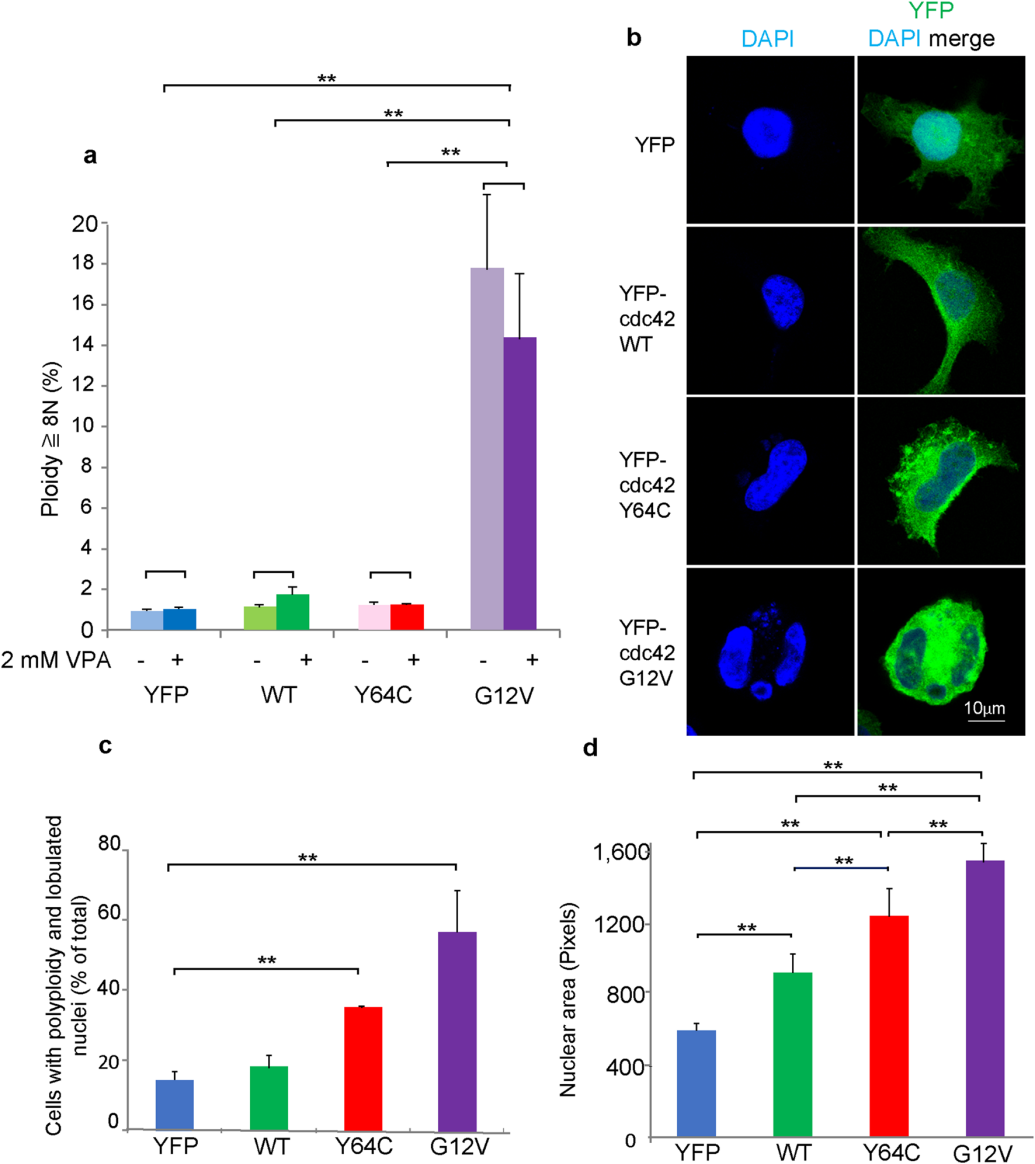
Cdc42 affected the nuclear morphological changes. (**a**) YFP-tagged Cdc42- or YFP-expressing MEG-01 cells were cultured for 3 days in the absence or presence of VPA. Cells were stained with PI and DNA ploidy was analyzed by FACS. The percentage of polyploid cells (8 N≧) is shown as the mean ± SD (n = 11) of four independent experiments. (**b**) Representative images of cells stained with DAPI cultured for 7 days in the presence of VPA. Scale bar: 10 μm (**c**) Cells with nuclear morphological changes were counted (108–731 cells per experiment) and reported as the percentage of total cells obtained from three independent experiments. Data are the mean ± SD. (**d**) Cells were cultured for 7 days in the presence of VPA. The nuclear area was analyzed in at least 80 cells from each cell type using Image Pro plus. Similar results were obtained from three independent experiments. Data are the mean ± SD. Significance was assessed by the Tukey–Kramer multiple comparison test, *P < 0.05, **P < 0.01.

Macrothrombocytopenia is one of the typical clinical phenotypes of TKS patients^11,14^. Next, we focused on the effects of Y64C in the process of thrombopoiesis. The MEG-01 cell line, which produces PLP, has been used in MK differentiation studies^24,28^. Upon the induction of differentiation by VPA, cells exhibit morphological changes, such as greater adherence and polyploidization, resulting in the production of PLP in a notch signaling-dependent manner^29,30^. To investigate whether Y64C affects polyploidy, all forms of YFP-Cdc42 were introduced into MEG-01 cells and FACS analysis was performed using PI staining. In contrast to previous findings^29^, polyploidy was not increased in the YFP control (YFP) or WT by the VPA treatment for 3 days. However, CA induced polyploidization in the absence and presence of VPA (Fig. 3a). As clear ploidy peaks were not detected by longer VPA treatment (data not shown), we investigated differences in the topological features of nuclei. DAPI staining was performed to analyze nuclear morphology and the area of the attached differentiating cells. In comparisons with YFP-expressing cells, nuclear morphological changes, including irregularly shaped or lobular nuclei, were prominent in CA-expressing cells, moderate in Y64C-expressing cells and slightly in WT-expressing cells (Fig. 3b,c, Supplemental Fig. 2 and 3). Similarly, the nuclear area significantly increased in CA- and Y64C-expressing cells compared with both WT- and YFP-expressing cells (Fig. 3d). On the other hand, WT-expressing cells had a similar area to YFP-expressing cells (Fig. 3d). Thus, Cdc42 activity promoted early MK differentiation, including nucleipolyploidization, in MEG-01 cells.

### The Y64C variant suppresses cytoplasmic extension during proplatelet formation

**Figure 4:**
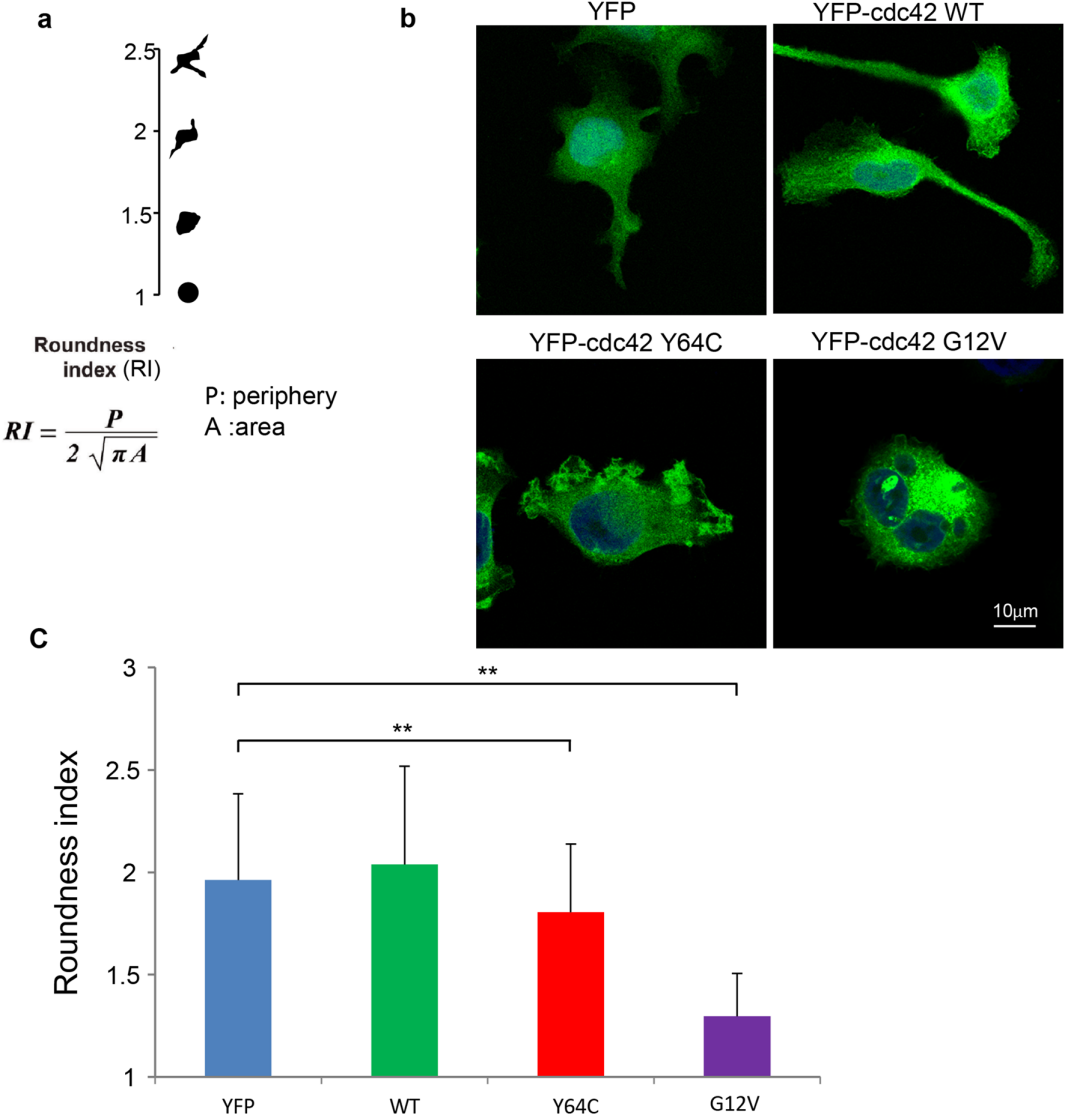
Y64C impaired the extension of MK protrusions. (**a**) To assess protrusions, the periphery (P) and area (A) of each cell were measured using software to calculate the roundness index (RI). The RI is a quantitative value that is proportional to the structural complexity of the cells regardless of their cytoplasmic mass. (**b**) Representative images of YFP-tagged Cdc42- or YFP-expressing MEG-01 cells cultured for 7 days in the presence of VPA. Scale bar: 10 μm (**c**) RI index of cells cultured for 7 days in the presence of VPA. Data are the mean ± SD (WT, n = 114, M, n = 130, CA, n = 165, YFP, n = 68) of three independent experiments. Significance was determined by the Tukey–Kramer multiple comparison test, **P < 0.01.

MK generates platelets by remodeling their cytoplasm into long proplatelet extensions^31^. To assess whether Y64C affects this process, we measured the circumference and area of attached cell bodies after MK differentiation. The roundness index (RI), which provides a quantitative value of the structural complexity of the cells, is calculated using a formula^32^ (Fig. 4a). The values start at RI = 1 for perfectly round cells. Y64C caused short irregular protrusions and significantly reduced RI in comparison with YFP, which led to long extensions (Fig. 4b and c). Furthermore, CA-expressing cells maintained a semi-round shape and had a markedly low RI value. However, no significant differences were observed between YFP-WT and YFP-Y64C. These results suggest that persistent activation of Cdc42 impairs the production of protrusions, which are necessary for proplatelet formation, and Y64C also prevented this process.

**Figure 5:**
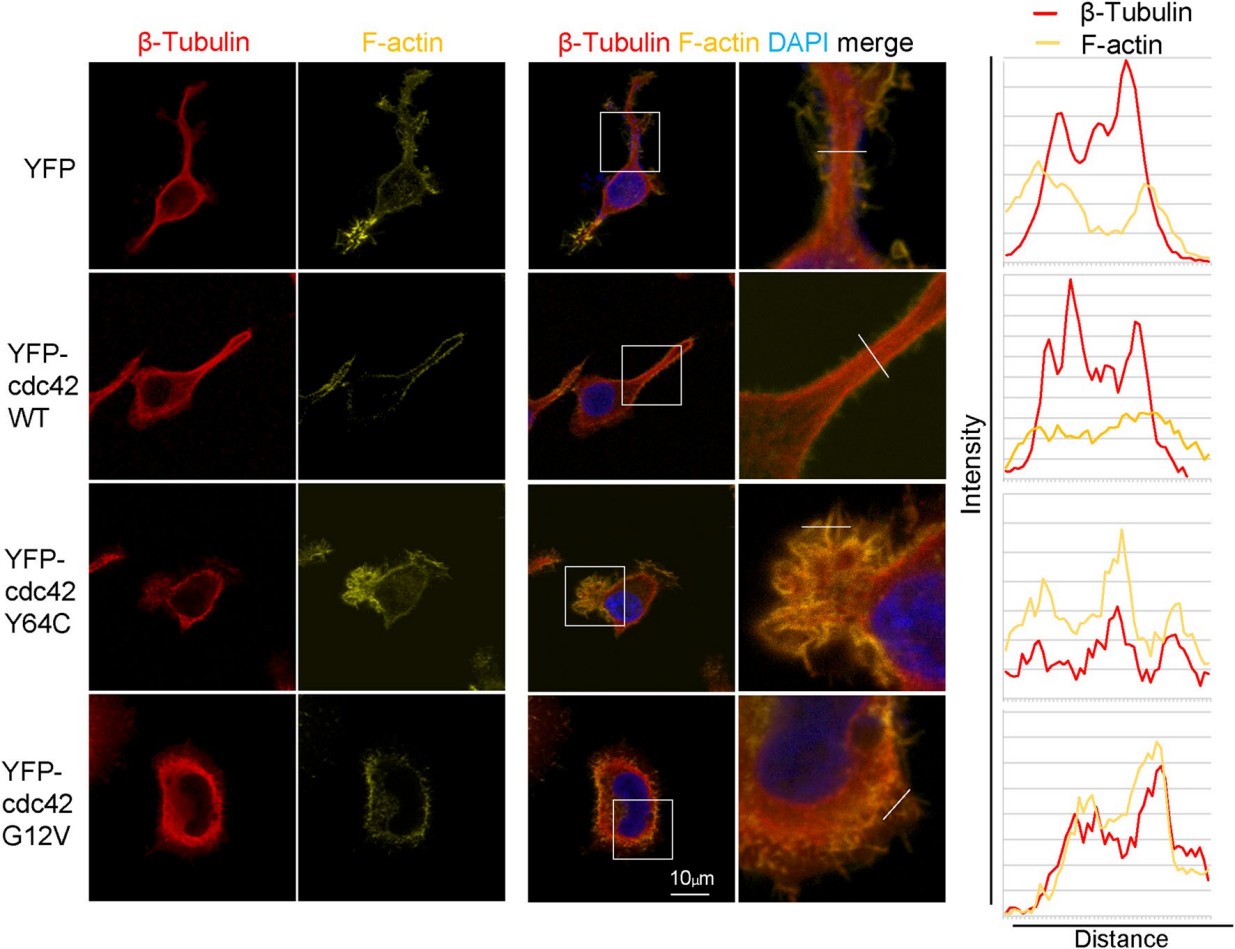
Abnormal colocalization of β-tubulin with F-actin in MK protrusions by Y64C expression. (**a**) Representative images of YFP-tagged cdc42- or YFP-expressing MEG-01 cells stained for F-actin (yellow), β-tubulin (red), and DAPI (blue) after 7-day culture in the presence of VPA. White squares indicate the magnified zone. (**b**) Line scans of F-actin (yellow) and β-tubulin (red) fluorescence intensity along the white line from a representative image from a. Scale bar: 10 μm.

MK-specific Rac and Cdc42-double knockout mice exhibit macrothrombocytopenia due to impaired microtubule formation^33^. To explore the cytoskeletal structure in proplatelet formation further, we performed fluorescent staining of β-tubulin and F-actin. As shown in Fig. 5a, the β-tubulin network at prolonged protrusions was straight and had a long fibrillary structure in YFP- and WT-expressing cells, whereas that in Y64C-expressing cells had immature short protrusions that were irregular and multidirectional with thick F-actin. On the other hand, the network of β-tubulin and F-actin was localized at the marginal ruffles in CA-expressing cells. Of note, measurement of fluorescence intensity in the protrusions of each cell demonstrated that F-actin and β-tubulin were colocalized in Y64C- and CA-expressing cells. However, such colocalization was not observed in WT-expressing cells (Fig. 5). The number of cells showing F-actin and β-tubulin colocalization in fibrillary structures were significantly higher in YFP-Y64C than in YFP (Supplemental Fig. 4). These observations reinforce the importance of Cdc42 in regulating actin and microtubule dynamics during proplatelet-like differentiation^21,33^.

### PLP production decreased in Y64C-expressing MEG-01 cells

**Figure 6:**
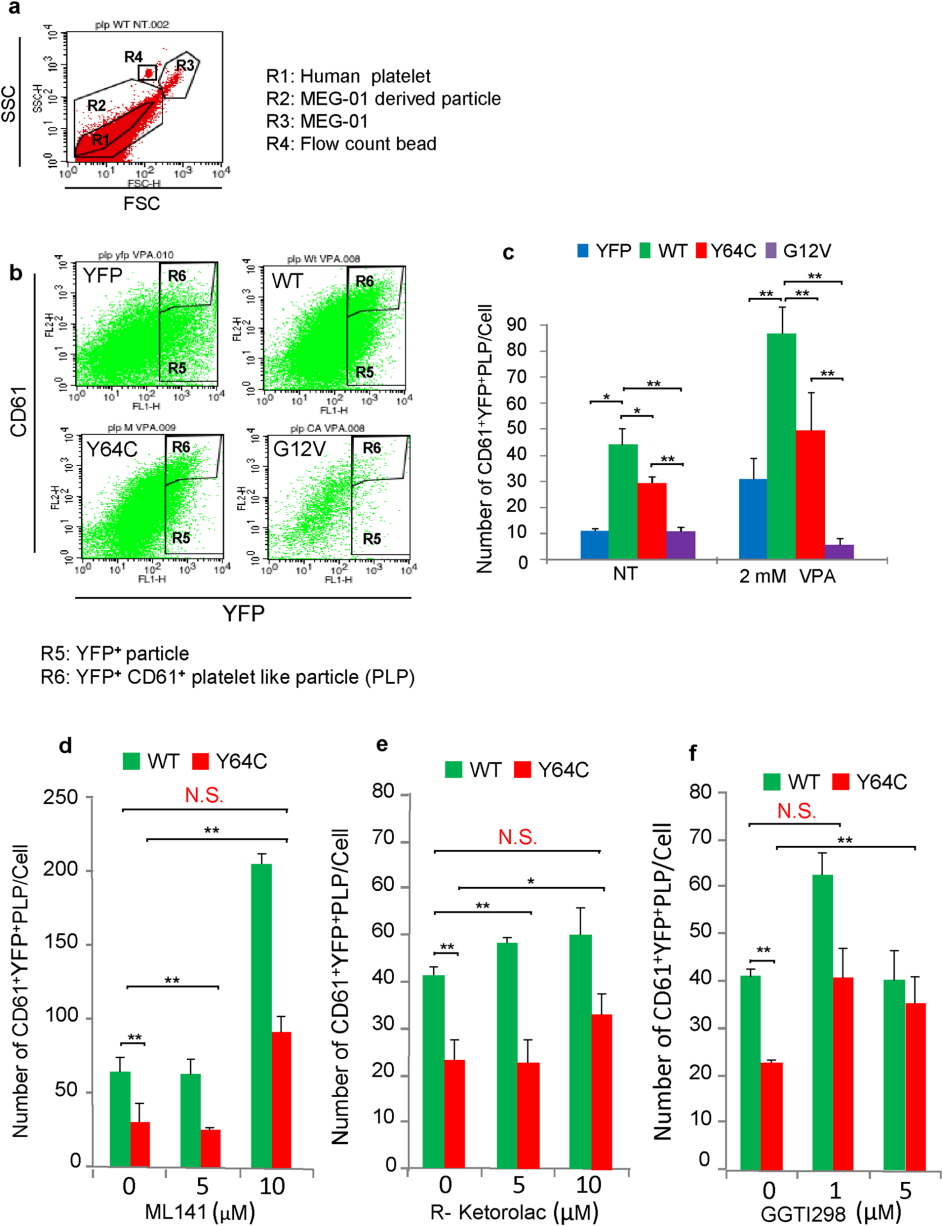
Reduced PLP production in Y64C was restored by inhibitors of Cdc42 activity or geranylgeranylation. (**a**) FACS plot panel shows the SSC and FSC properties of particles derived from YFP-expressing MEG-01 cells treated with VPA for 12 days. The R1 gate is the platelet population in peripheral blood determined by CD61 expression. The R2 gate is used to detect PLP derived from VPA-treated MEG-01 cells after 12-day culture. R3 and R4 gates are MEG-01 cells and flow count beads, respectively. (**b**) Representative FACS plot of R2-gated particles derived from YFP-tagged Cdc42- or YFP-expressing MEG-01 cells cultured for 12 days in the presence of VPA. Particles were further gated to assess the expression of YFP (R5). The R6 (YFP^+^CD61^+^) gate was used to detect PLP. (**c**) The number of PLP from a single cell in the absence or presence or VPA at 12 days. Data are the mean ± SD (n = 3). The results are representative of three independent experiments. (**d**, **e** and **f**) The numbers of PLP from a single cell cultured in the presence of VPA and ML141 (5–10 μM) (**e**), R-Ketorolac (5–10 μM) or (**f**) GGTI-298 (1–5 μM). Data are the mean ± SEM (n = 3). The results are representative of three independent experiments. Significance was determined by the Bonferroni/Dunn multiple comparison test, *P < 0.05, **P < 0.01. N.S.: not significant (*P* > 0.05).

We next investigated whether Y64C affects PLP production. FACS analysis was performed to count YFP- and CD61- double-positive PLP (Supplemental Fig. 5) based on size and complexity (FSC and SSC) similar to peripheral blood platelets (Fig. 6a,b). We confirmed that VPA treatment promoted PLP production in YFP, WT- and Y64C-expressing cells (Fig. 6b,c). On the other hand, CA did not respond to VPA. PLP production was significantly lower in CA-expressing cells, and modestly reduced in Y64C-expressing cells in comparison with YFP- or WT-expressing cells in the presence or absence of VPA (Fig. 6b,c). Collectively, this suggests that Y64C promotes Cdc42 activity and membrane localization, resulting in impaired PLP production mimicking a low platelet count in TKS patients.

### Cdc42-specific inhibitors and GGTI recovery reduced PLP production in Y64C-expressing cells

**Figure 7:**
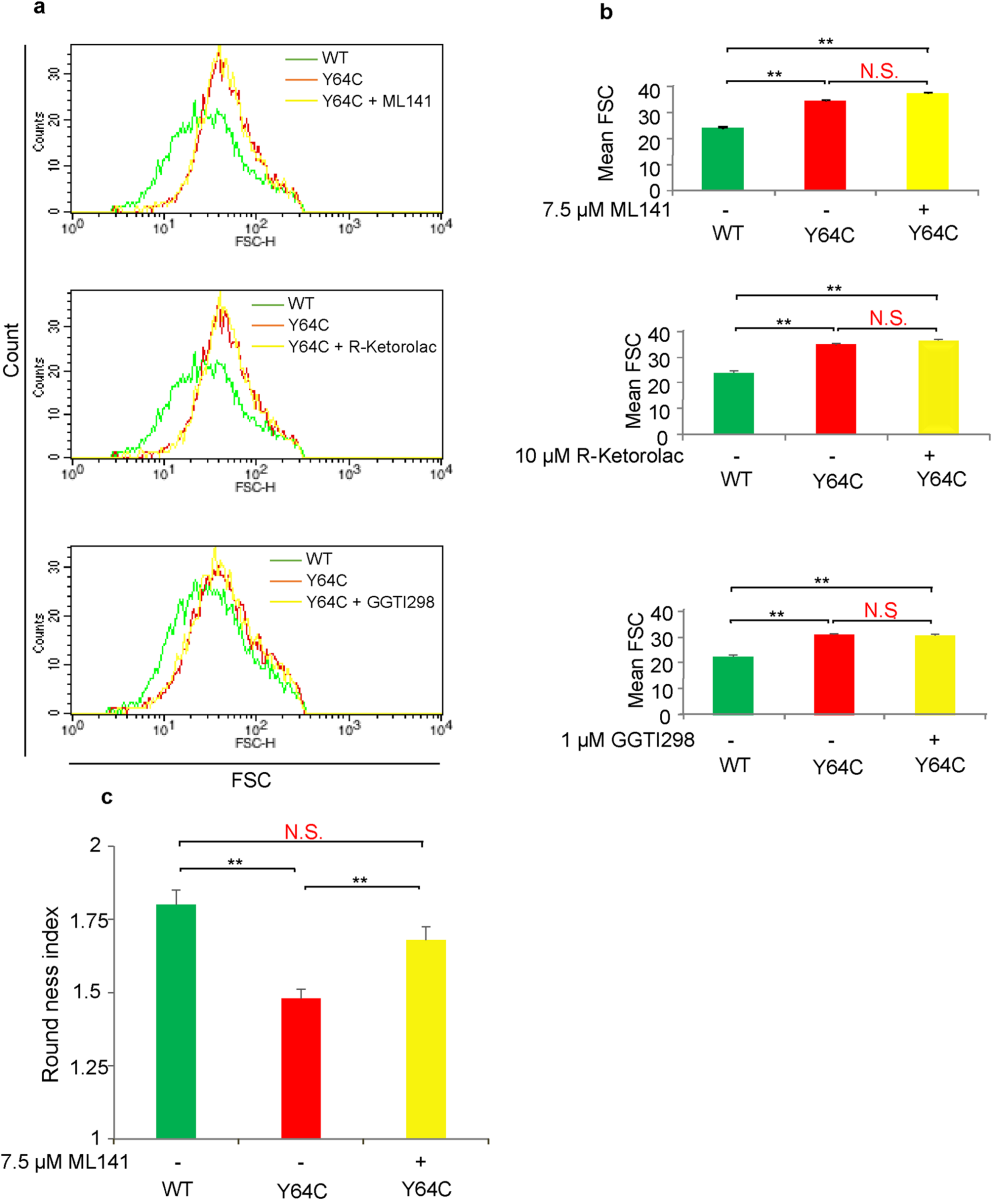
ML141 improved the extension of MK protrusions, but not PLP size, in Y64C-expressing MEG-01 cells. (**a**) Representative FACS histogram plot of PLP size using FSC as the size parameter. YFP-tagged WT- or Y64C-expressing MEG-01 were treated with VPA for 12 days in the absence or presence of ML141, R-ketorolac or GGT-I298. (**b**) Geomeans of FSC are the mean ± SEM (n = 4). The results are representative of three independent experiments. Significance was determined by the Bonferroni/Dunn multiple comparison test (**c**) Cells were cultured for 7 days in the presence of VPA. ML141 was added on day 6 for 24 h. RI values are the mean ± SD (WT, n = 57, M, n = 69, ML141 treated M, n = 53). The results are representative of three independent experiments. Significance was assessed by the Tukey–Kramer multiple comparison test, **P < 0.01, N.S.: not significant *P * > 0.05.

We next attempted to restore PLP production using ML141 or R-Ketorolac, specific inhibitors of Cdc42^34^. In addition to these activity-specific inhibitors, GGTI-298, a derivative of the geranylgeranyl transferase inhibitor, which inhibits membrane localization of Ras and Rho GTPase^35^, was expected to suppress the membrane localization of Y64C. As expected, treatment with both ML141 (10 μM) and R-ketorolac (10 μM) promoted PLP production in WT-expressing cells (Fig. 6d,e). Of note, reductions in PLP production by Y64C expression were increased in the presence of ML141 and restored to the same level as in non-treated WT controls (Fig. 6d). R-Ketorolac partially attenuated the inhibitory effects of Y64C (Fig. 6e). In addition, GGTI-298 (1 μM) improved PLP production in Y64C-expressing cells to the same level in WT-expressing cells without GGTI-298 treatment (Fig. 6f). This suggests that reduced PLP production by Y64C was ameliorated by Cdc42-specific inhibitors and GGTI-298. As mutations in the switch II domain, including Y64C^6,14,36^, or in the C-terminal region cause macrothrombocytopenia^37^, we next assessed whether the size of PLP was influenced by Y64C using FACS analysis. PLP from Y64C-expressing cells were significantly larger than those from YFP and WT based on increased FSC (Fig. 7a, Supplemental Fig. 6a and b). Next, to examine whether the increase in PLP size was inhibited, the PLP size was analyzed in the presence of the above inhibitors (Fig. 7a,b). However, these inhibitors which restored the PLP number did not affect their size, although proplatelet differentiation indices calculated by RI improved to the same level as in WT-expressing cells (Fig. 7c). Thus, inhibition of the activity or localization of Y64C by inhibitors restored proplatelet formation and PLP production in MEG-01 cells.

## Discussion

Thrombocytopenia is a common pathology of missense mutations in the switch II region of Cdc42, which mediates its binding to effectors and regulators^6,13,14^. TKS manifesting with macrothrombocytopenia is caused by Y64C in this region. To better understand how Y64C affects thrombopoiesis resulting in macrothrombocytopenia, we used the MEG-01 cell line, which generates PLP via MK differentiation.

In this study, we demonstrated that Y64C-expressing MEG-01 cells had reduced PLP production with a larger PLP size than WT-expressing cells. This phenotype coincides with macrothrombocytopenia in TKS patients. Using this model, we found that Y64C overexpression suppressed proplatelet extension in MEG-01 cells. We also demonstrated the colocalization of F-actin and β-tubulin in short and irregular protrusions in Y64C expressing cells, but in WT-expressing cells. Thus, Y64C may impair proplatelet maturation associated with disorganized cytoskeletal rearrangement of both F-actin and microtubules, resulting in concomitant PLP secretion reduction and increased PLP size. Importantly, we confirmed that the association with Rho-GDI was suppressed by Y64C, and this variant facilitated membrane localization and GTP binding. The ectopic Y64C activity provides a plausible explanation for both the reduced PLP production in MEG-01 cells and macrothrombocytopenia in TKS patients. Supporting our hypothesis, we demonstrated the suppression of Cdc42 activity and membrane localization using inhibitors that restored PLP production, although the PLP size was not affected by these inhibitors.

Y64C exhibited much higher activity than WT in vitro, consistent with a previous report^16^. On the other hand, the Y64C allele had hypomorphic effects in a C. elegans model^36^. This discrepancy may be related to the location of the mutation within the switch II motif, which mediates the interaction with its regulators and downstream effectors. Indeed, consistent with a previous report^6^, Y64C did not associate with its downstream effectors Pak1/2 and N-WASP. Bone marrow-specific PAK2 deletion is related to macrothrombocytopenia and negatively regulates polyploidy^38^. As shown in Fig. 3, overexpression of Y64C accelerated nuclear maturation in MEG-01 cells. This suggests that Pak2 signaling is impaired by Y64C during MK differentiation and platelet production in MEG-01 cells. On the other hand, CA-expressing cells had markedly higher ploidy. Roy et al. reported that the RhoA/ROCK/NMIIA pathway negatively regulated endomitosis^39^ and Pleines et al. demonstrated that Rac1 and Cdc42 were not necessary for polyploidy^33^. The excessive Cdc42 activity may influence the balance between Rho-GTPases. Further investigation is necessary to elucidate the role of Cdc42 in polyploid formation in MEG-01 cells.

Several investigators reported that microtubule reorganization is important for thrombopoiesis^40–42^ and mutation of β-tubulin is associated with macrothrombocytopenia^43,44^. The present results indicate that Cdc42 signaling affected tubulin organization, and the hyperactivation of Cdc42 disorganized microtubules and the actin cytoskeleton. The exact regulatory mechanism of microtubule dynamics by Cdc42 remains unclear in MEG-01 cells. However, IQGAP1, one of the Cdc42 downstream effectors, has been shown to directly or indirectly bind to microtubules and regulate microtubule organization in vitro^45,46^. IQGAP1 expression levels were found to be reduced in Rac1/Cdc42-lacking megakaryocytes in mice^33^. Formins which were initially reported to play a role in actin nucleation and elongation under Cdc42 signaling bridged microtubules and actin filaments in axonal growth cones^47^. Moreover, the cdc42 effector LIMK coordinated microtubule stability and actin polymerization in human endothelial cells^48^. These findings support our hypothesis that the higher activity of the Y64C variant disturbs crosstalk between microtubules and F-actin, which is necessary for proplatelet extension. Mutations in the *MYH9* gene cause macrothrombocytopenia with a number of other clinical conditions^49,50^. MK derived from MYH9-related diseases exhibit defective proplatelet formation due to excess actomyosin contractility in spreading MK^51^. PAK has been shown to promote the phosphorylation of myosin light chains in endothelial cells^52^ and NIH3T3^53^. Aberrant PAK signaling in Y64C-expressing MEG-01 cells may affect the regulation of actomyosin, which is required for the differentiation of PLP.

In conclusion, this study confirmed that Y64C alters the activity and localization of Cdc42, thereby impairing the assembly of functional F-actin and microtubules for proplatelet extension, resulting in a reduced number of PLP. Importantly, these defects were ameliorated by inhibitors that regulate either Cdc42 activity (ML141 and R-ketorolac) or cellular localization (GGTI-298). Among these drugs, R-Ketorolac, an FDA-approved drug, significantly affected the survival of ovarian cancer patients in a P0 clinical trial^54^. This may provide a pharmacological approach not only to macrothrombocytopenia, but also to other symptoms, including immune dysregulation^12,14,15^, in TKS patients. In addition, MK and platelet differentiation models using MEG-01 cells may be useful to screen for new pharmacotherapeutic agents for thrombocytopenia.

## Supplementary Information

Supplementary Information.

## Data Availability

All data generated or analyzed during this study are included in this article.
