# Supplementary material for: Macrothrombocytopenia of Takenouchi-Kosaki syndrome is ameliorated by CDC42 specific- and lipidation inhibitors in MEG-01 cells
Source: Sci Rep. 2021 Sep 9;11:17990. doi: 10.1038/s41598-021-97478-y (PMC8429552; doi:10.1038/s41598-021-97478-y)
Supplement: Supplementary file 1 — Supplementary Information. [file 41598_2021_97478_MOESM1_ESM.pdf]

Macrothrombocytopenia of Takenouchi-Kosaki syndrome is ameliorated by CDC42 specific- and lipidation inhibitors in MEG-01 cells

Etsuko Daimon[1†], Yukinao Shibukawa[1†], Suganya Thanasegaran[1], Natsuko Yamazaki[1] and Nobuhiko Okamoto[1,2, *]

[1]Department of Molecular Medicine, [2]Department of Medical Genetics, Research Institute, Osaka Women's and Children's Hospital, 840 Murodo-cho, Izumi, Osaka 594-1101, Japan

[†]Contributed equally to this work
[*] To whom all correspondence should be addressed.

Department of Molecular Medicine
Research Institute, Osaka Women's and Children's Hospital
840 Murodo-cho, Izumi, Osaka 594-1101, Japan
Tel: 81-725-56-1220
Fax: 81-725-57-3021
E-mail: genetics@wch.opho.jp

Supplemental Fig. 1

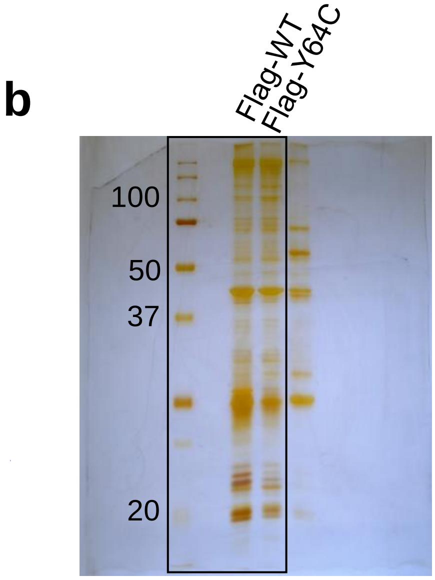

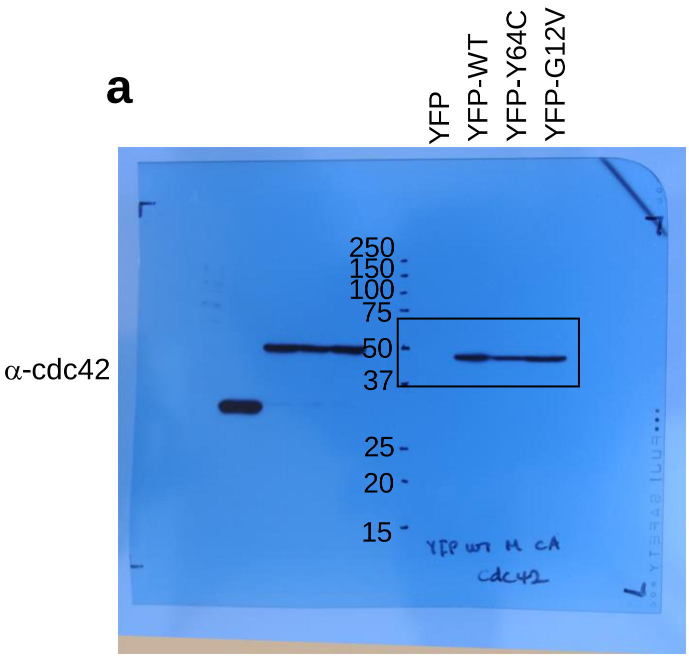

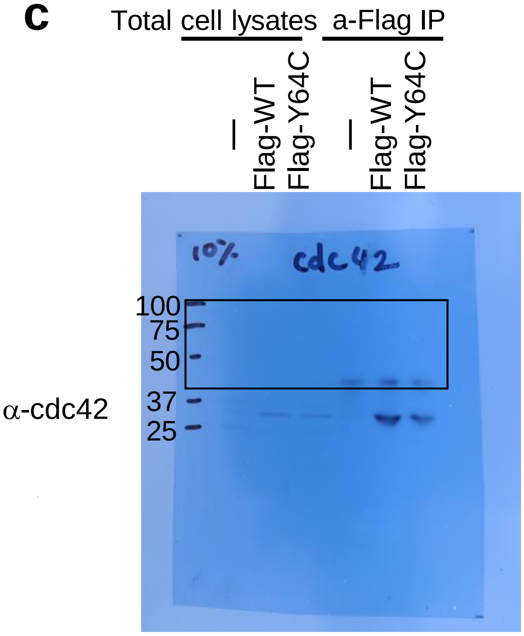

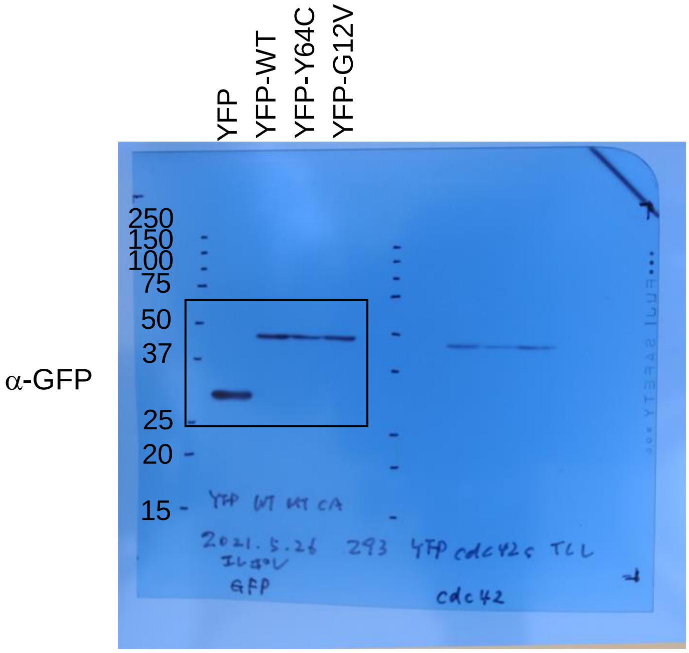

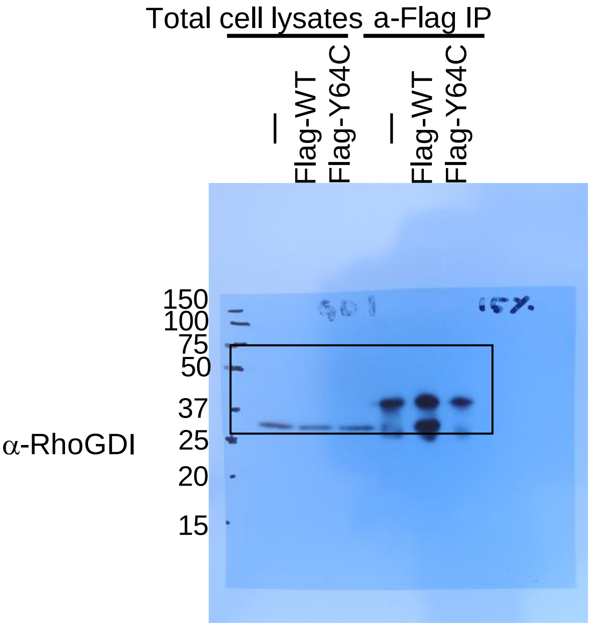

Supplemental Fig. 1 Full blot and gel images
Full blot images for Fig. 1a (a), Fig. 1d (c), Fig. 2a (d), Fig. 2b (e), and Fig. 2c (f). Silver staining gel image for Fig. 1c (b). Box shows selected band for the main Figure.

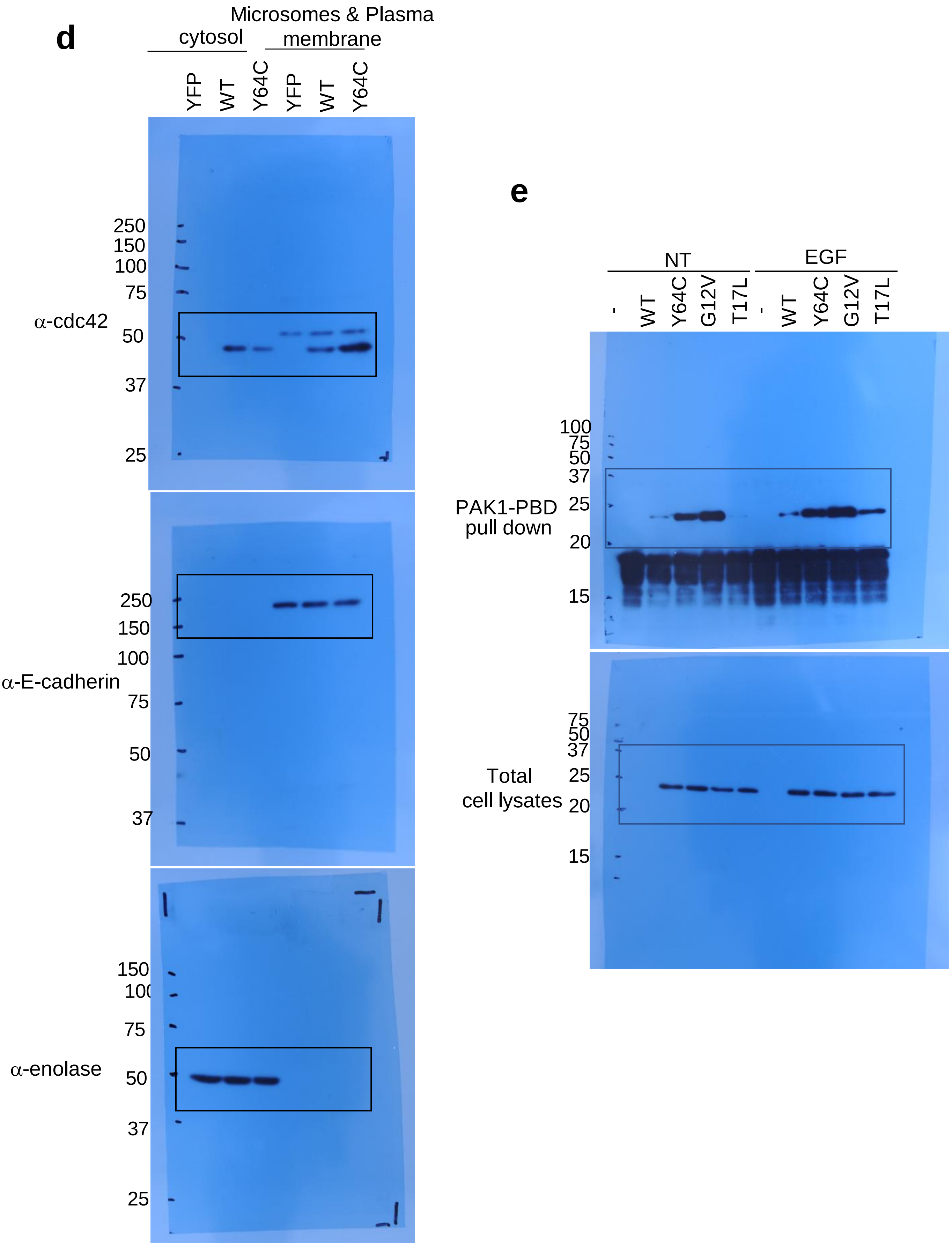
d
cytosol
Microsomes & Plasma membrane
YFP
WT
Y64C
YFP
WT
Y64C
α-cdc42
250
150
100
75
50
37
25
α-E-cadherin
250
150
100
75
50
37
α-enolase
150
100
75
50
37
25
e
NT
EGF
-
WT
Y64C
G12V
T17L
-
WT
Y64C
G12V
T17L
PAK1-PBD pull down
100
75
50
37
25
20
15
Total cell lysates
75
50
37
25
20
15

f

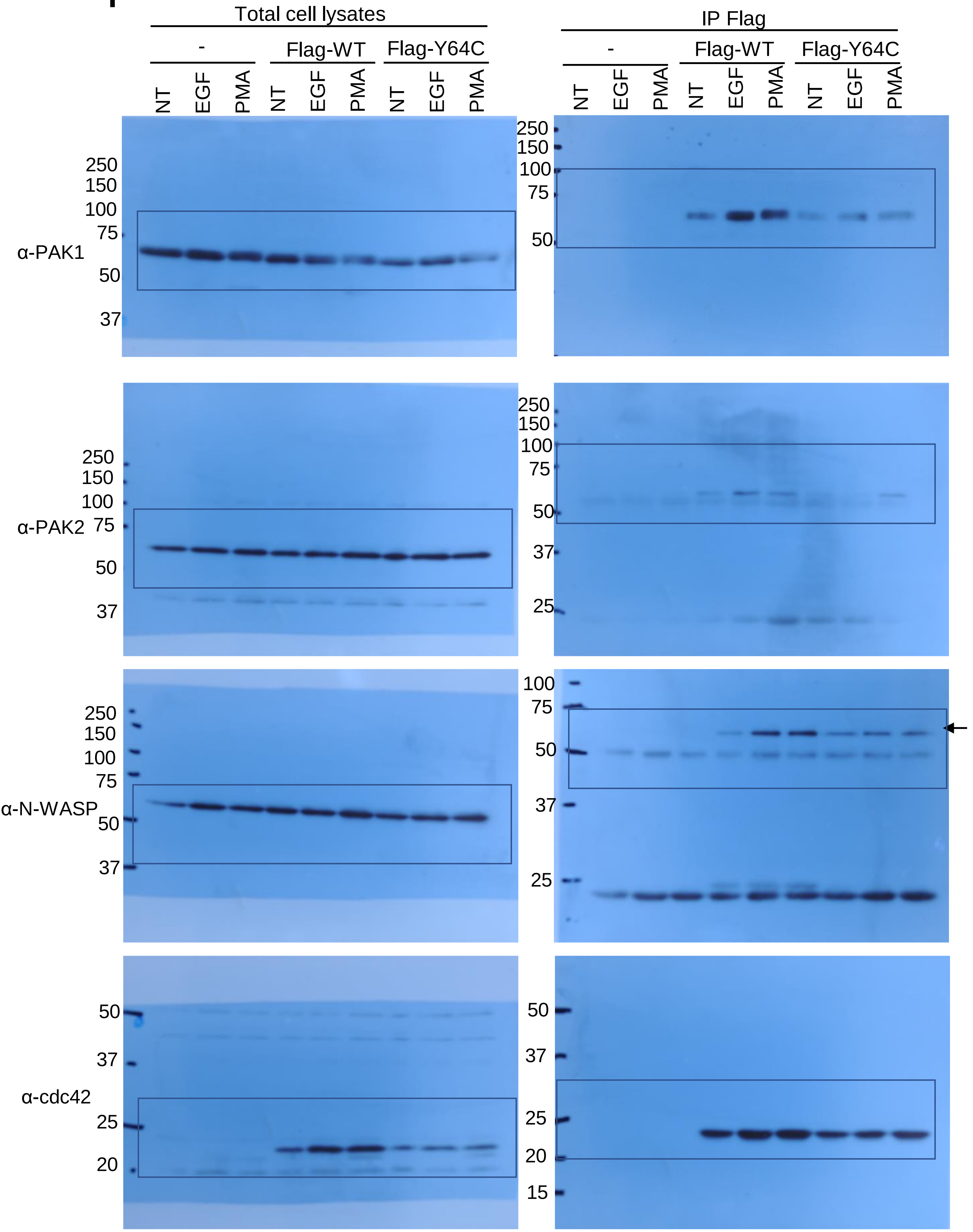
Total cell lysates
-
Flag-WT
Flag-Y64C
NT
EGF
PMA
IP Flag
α-PAK1
α-PAK2
α-N-WASP
α-cdc42
250
150
100
75
50
37
25
20
15

Supplemental Fig. 2

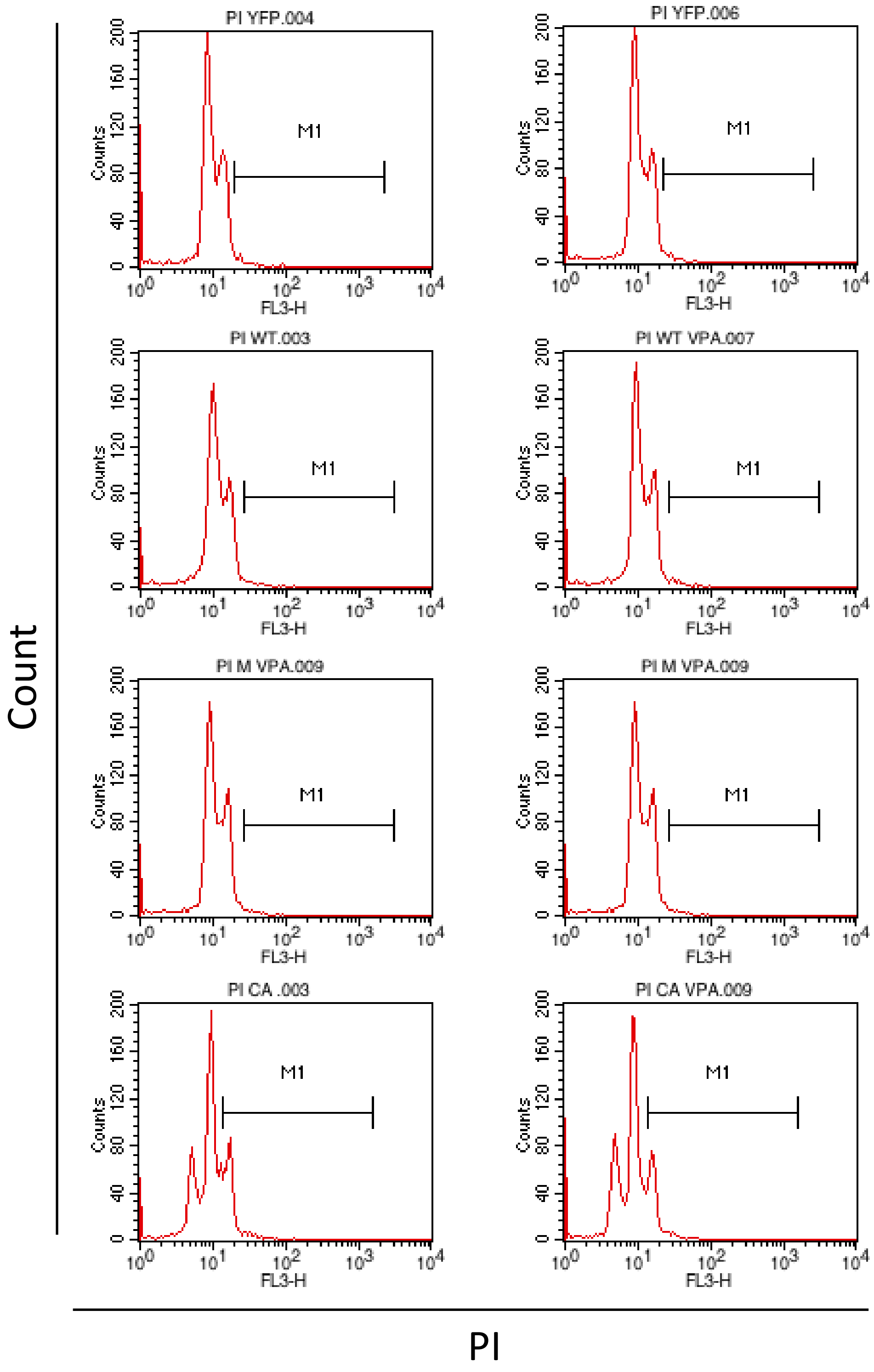

Supplemental Fig. 2 Representative charts of PI intensity by FACS
The X axis indicates the signal intensity of PI, which reflects the nuclear DNA content. M1 indicates polyploid cells (8 N≧).

Supplementary Fig. 3

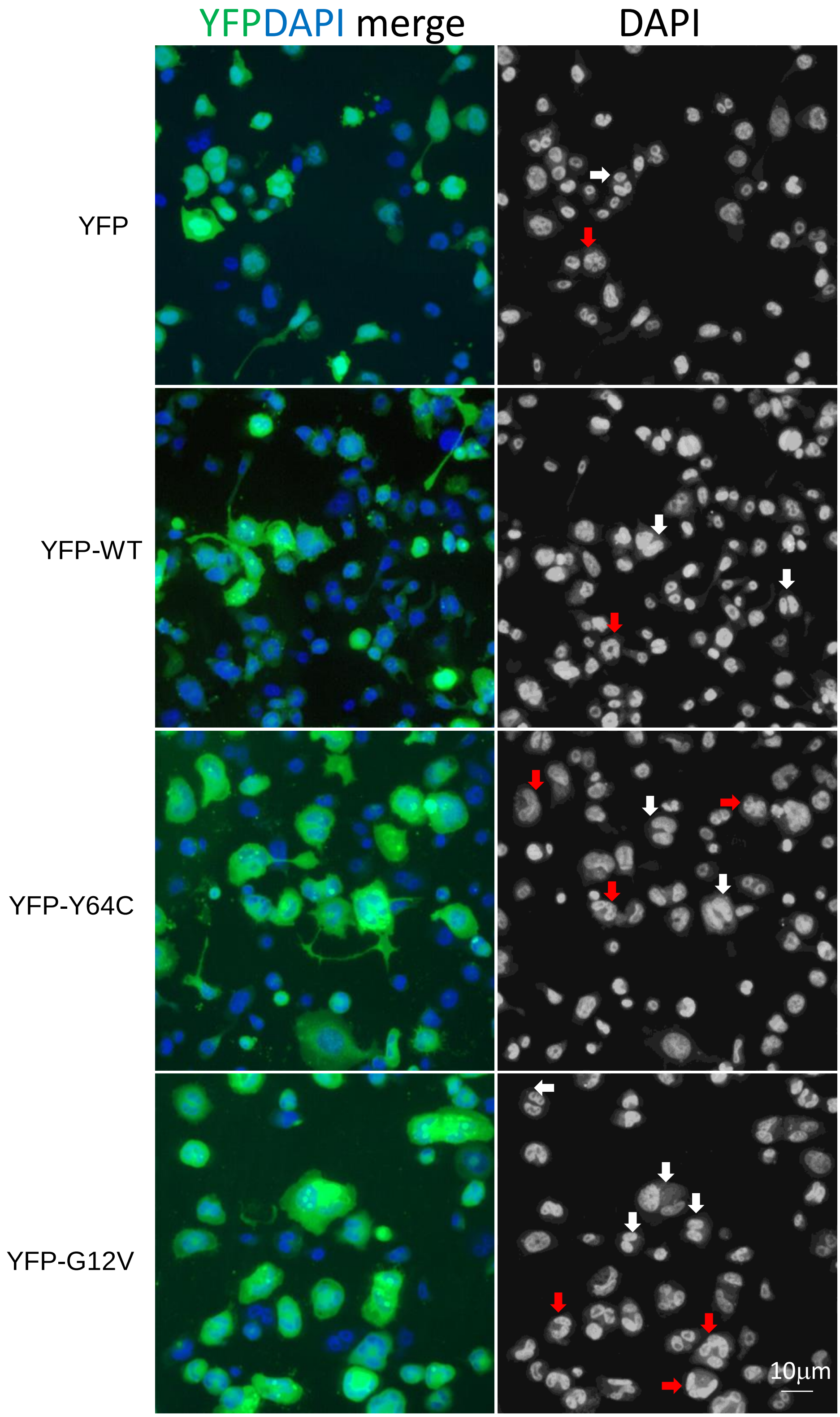

Supplemental Fig. 3 Representative images of cells stained with DAPI to quantify the nuclear area and morphology

The nuclear area was analyzed by software and cells with nuclear lobulation and ploidy were counted from at least 5 randomly selected photographs. The white arrow indicates ploidy cells. The red arrow indicates a cell with nuclear lobulation.

Supplemental Fig. 4

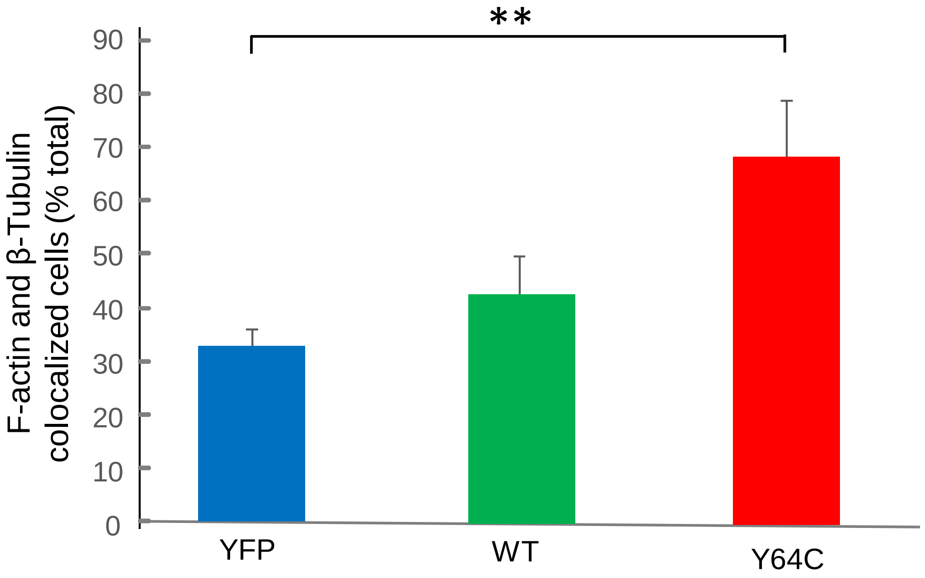

Supplemental Fig. 4 Quantitation of cells showing the colocalization of F-actin and β-tubulin

Line scans were performed at the fibrillar structures that formed in the longest protrusions. At least 20 cells were analyzed. The percentage of cells displaying the colocalization of F-actin and β-tubulin is shown as the mean ± SD of three independent experiments. Significance was assessed by the Bonferroni/Dunn's multiple comparison test, **, $P < 0.01$.

Supplemental Fig. 5

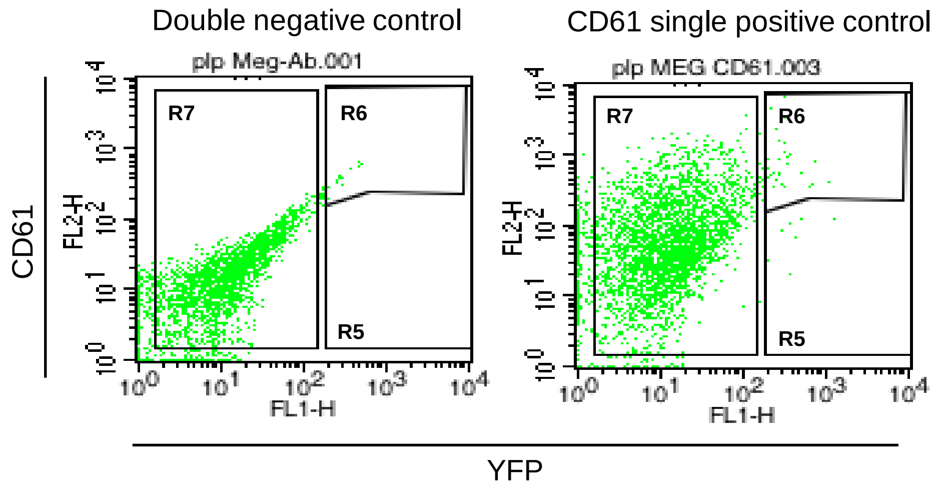

Supplemental Fig. 5 Representative images of PLP derived from MEG-01 cells stained with or without an anti-CD61 antibody
The R5 gate was assessed by the intensity of YFP$^-$ emitted by PLP derived from MEG-01 treated with (Single positive control) or without (double negative control) an anti-CD61 antibody. R5: YFP$^+$ particle, R6: YFP$^+$CD61$^+$ PLP, R7: YFP$^-$ particle.

Supplemental Fig. 6

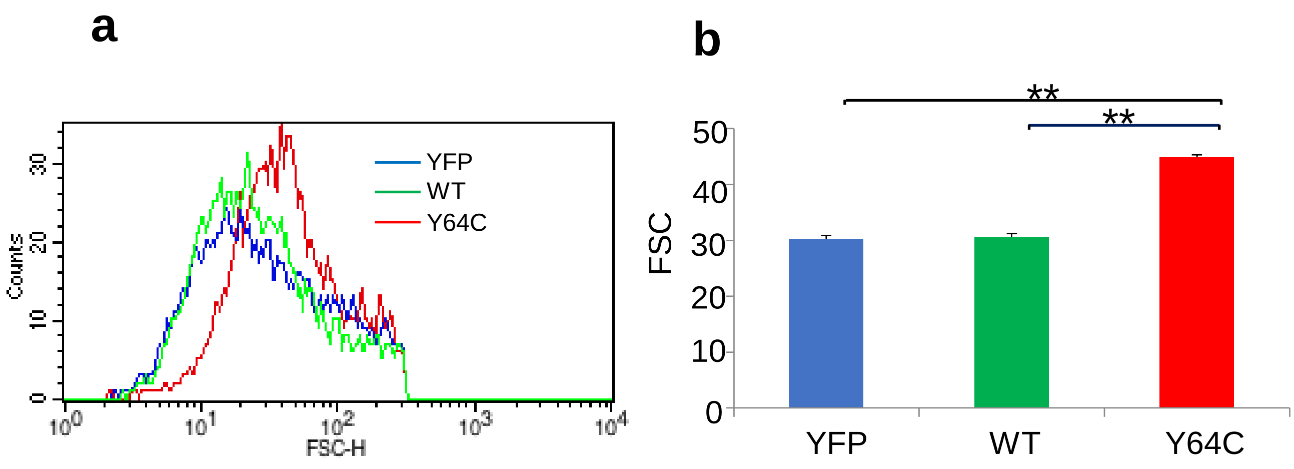

Supplemental Fig.6 PLP derived from Y64C were larger than those from YFP or WT
(a) Representative PLP sizes were shown in a FACS histogram using FSC as the size parameter. (b) Geomeans of FSC were shown as the mean $\pm$ SEM (n=4). Similar results were obtained from three independent experiments. Significance was assessed by the Bonferroni/Dunn's multiple comparison test, **, $P < 0.01$.
